# The impact of teach-back on patient recall and understanding of discharge information in the emergency department: the Emergency Teach-Back (EM-TeBa) study

**DOI:** 10.1186/s12245-020-00306-9

**Published:** 2020-09-24

**Authors:** Mandhkani Mahajan, Janine Alida Hogewoning, Jeroen Joseph Antonius Zewald, Margreet Kerkmeer, Mathilde Feitsma, Daphne Annika van Rijssel

**Affiliations:** 1 Department of Emergency Medicine, Reinier de Graaf Hospital, P.O. Box 5011, 2600 GA Delft, The Netherlands; 2 Science Department, Reinier de Graaf Hospital, Reinier Academy, P.O. Box 5011, 2600 GA Delft, The Netherlands

**Keywords:** Patient communication, Comprehension and recall, Teach-back, Patient education

## Abstract

**Background:**

Previous research has demonstrated that patients leaving the emergency department (ED) have poor recall and understanding of their discharge information. The teach-back method is an easy technique that can be used to check, and if necessary correct, inaccurate recall. In our study, we aimed to determine the direct and short-term impact of teach-back as well as feasibility for routine use in the ED.

**Methods:**

A prospective cohort study in an urban, non-academic ED was performed which included adult patients who were discharged from the ED with a new medical problem. The control group with the standard discharge was compared to the intervention group using the teach-back method. Recall and comprehension scores were assessed immediately after discharge and 2–4 days afterward by phone, using four standardized questions concerning their diagnosis, treatment, follow-up care, and return precautions.

**Results:**

Four hundred eighty-three patients were included in the study, 239 in the control group, and 244 in the intervention group. Patients receiving teach-back had higher scores on all domains immediately after discharge and on three domains after 2–4 days (6.3% versus 4.5%). After teach-back, the proportion of patients that left the ED with a comprehension deficit declined from 49 to 11.9%. Deficits were most common for return precautions in both groups (41.3% versus 8.1%). Teach-back conversation took 1:39 min, versus an average of 3:11 min for a regular discharge interview.

**Conclusion:**

Teach-back is an efficient and non-time-consuming method to improve patients’ immediate and short-term recall and comprehension of discharge information in the ED.

## Background

A comprehensive discharge interview by healthcare providers is of utmost importance in the setting of the emergency department (ED). This information should at least include the diagnosis, treatment (including medication and self-care), follow-up consultations, and return precautions [1, 2]. Incomplete or ineffective discharge interviews can lead to health hazards such as nonadherence to prescribed medication, inappropriate homecare, and failure to act on return precautions [3–5]. This in turn may lead to a complicated course of disease and unnecessary return ED visits [6, 7]. Unfortunately, previous studies have shown that in 17–42% of the time the provider delivers insufficient information [8–10], and the majority of patients (41–78%) leave the ED with comprehension deficits [8, 9, 11–15]. Information was most lacking in post-ED care, including home care and return instructions [11, 12, 15]. In addition, most patients are unaware of their own lack of knowledge [12, 16]. Though traditionally patients with low health literacy or advanced age are thought to be at risk for comprehension deficits, many of these studies included a general ED population and did not discriminate in education level or socioeconomic status.

Different methods have been investigated to address this problem. Written instructions can be an option, but most patient information leaflets outline general problems and are not personalized to the patients’ specific medical problem. In addition, they are of limited use in case of low health literacy [17]. Electronic information is another option, but these usually are not personalized either and can be expensive [18, 19]. Promising results have been shown for the teach-back method [15, 20–23]. This approach is an evidence-based technique to verify not only patient recall, but also their understanding by asking them to repeat the given discharge information in their own words. The healthcare provider can then correct inaccurate information, if necessary [23].

To our knowledge, only two studies have studied the effect of using the teach-back method in the ED setting [15, 20]. Griffey et al. (2015) showed an improvement in comprehension of post ED care for patients with limited health literacy, whereas Slater et al. (2017) showed a positive effect in all ED patients, regardless of age and education. Though showing promising results, they did not study the delayed recall of information and did not specify which patient groups were included, making it difficult to extrapolate the results to non-academic hospitals with different patient and ED characteristics. Furthermore, the studies included both patients with existing and new medical problems or used teach-back as a supplement to written instructions. Lastly, no study has ever looked at the feasibility of using this technique in an ED setting.

The aim of this study was to analyze the isolated effect of the teach-back method on both direct and delayed recall in a general ED population and to assess the feasibility of routine use in the ED.

## Methods

### Study design and setting

A single-center, prospective cohort study was conducted between June 2018 and January 2019 in an urban, non-academic hospital (Reinier de Graaf Hospital in Delft, The Netherlands) with over 33,000 ED visits per year. The regional and local hospital ethics board approved this study.

### Study population

Inclusion criteria were patients ≥ 18 years being discharged from the ED with a new diagnosis. Exclusion criteria were a language barrier (non- or insufficient Dutch-speaking patients) or a diagnosis of altered mental status (dementia, delirium, coma), intoxication, or a psychiatric disorder with mental impairment. Researchers included patients during day, evening, and weekend shifts. Eligibility of patients was assessed by screening the electronic patient records and, if needed, consulting with the responsible healthcare provider. All patients provided verbal and written informed consent before enrolling.

### Study protocol

Two consecutive cohorts of patients were compared, the first being the control group and the second the intervention group. The first cohort was included from June to November 2018. In this group, recall and comprehension were scored after standard discharge. The second cohort was included from November 2018 to January 2019, where patients were scored after the teach-back method was added.

In the control group, the researcher was present at discharge to listen to the content of the discharge information given by the physician. After discharge, patient recall was scored by asking four questions across the following domains: diagnosis (“What was the medical explanation for your complaints?”), treatment (“What is the treatment?”), follow-up care (“What are the follow-up appointments?”), and return instructions (“Which symptoms are a reason for you to revisit a doctor?”). If a domain was not discussed, the question related to that domain was not asked. A four-level scale was used (1 = none, 2 = minimal, 3 = partial, 4 = complete comprehension). The maximum obtainable score was 16, and the minimum 4. After 2–4 days, all patients were rescored by telephone by asking them the same four questions.

After completing the first cohort, 1 week was used to inform all nurses about the teach-back method via presentations, information letters, and pocket cards. They were instructed to ask patients to restate in their own words what they had been told about the four domains as mentioned earlier. If patients were unable to recapitulate what was told, the nurse was instructed to repeat the information again in different words. They were also directed to use understandable language with open questions to prevent the patient of feeling interrogated. We chose to use nurses to conduct the teach-back interview as they tend to be seen as more accessible by patients and therefore might make patients feel less embarrassed to answer questions they might not know the answer to.

After the training period, inclusions for the second cohort started. In addition to the doctor and researcher, the nurse was also present at discharge. After the doctor left, patients received the teach-back interview by their nurse. This was followed by the same recall interview as in the first cohort done by the researcher. These patients were also phoned after 2–4 days to re-score them.

### Outcome measures and data collection

The primary outcome was the difference in the total score of the discussed domains between the control and intervention group at baseline. Secondary outcomes were the differences in scores for each domain, the follow-up scores, and the longitudinal scores. The percentage of discharged patients with complete and incomplete comprehension was calculated at both baseline and follow-up. We also recorded how often no information was provided to the patient per domain. To assess feasibility, the duration of the teach-back conversation in comparison with the duration of discharge was measured.

Additional data about age, gender, and highest level of patient education was collected as baseline characteristics. Treating specialty, complexity of the medical problem (low, medium, or high), presence of a partner during discharge (yes/no), and time of discharge conversation (peak hours yes/no) were registered. Subsequently, the data was entered in Castor EDC (version 2019.1.3) using the hospital-related patient identification number. The patient identification number was blinded and only visible to the involved researchers.

### Statistical analysis

Based on a power of 0.90 and an alfa of 0.05, 191 patients per study group needed to be included to demonstrate an effect size of 0.30. To account for potential loss at follow-up, the aim was to include 200 patients per study arm. Sample size calculation was performed with GPower (Version 3.1, Heinrich Heine University, Düsseldorf).

Continuous data were presented as mean values with standard deviations or medians with interquartile range (IQR) and nominal variables as absolute numbers with frequencies. Outcomes were compared between both groups using the one-way ANOVA (Welch) and chi-square test for continuous and nominal variables, respectively. Comparisons within each cohort of the baseline and follow-up time points were performed using the paired *t* test. A sensitivity analysis was performed to determine the robustness of results. To correct for possible confounding, a linear regression analysis was performed.

Statistical analysis was conducted using SPSS version 25.0. A *p* value < 0.05 was determined to be statistically significant.

## Results

### Patient sample baseline

**Fig. 1:**
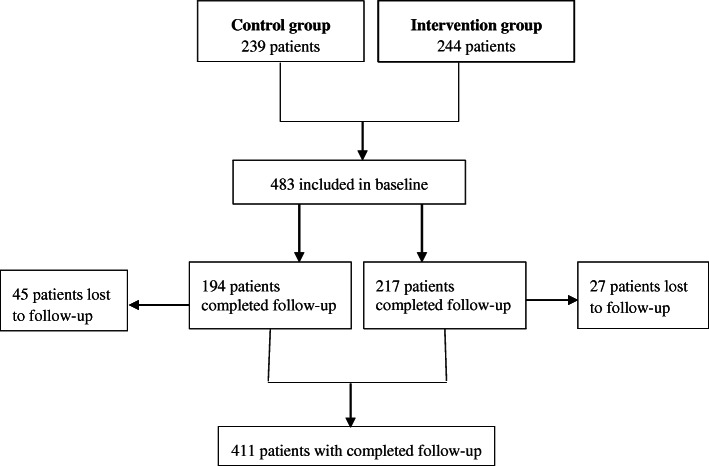
Patient flow chart

**Fig. 2:**
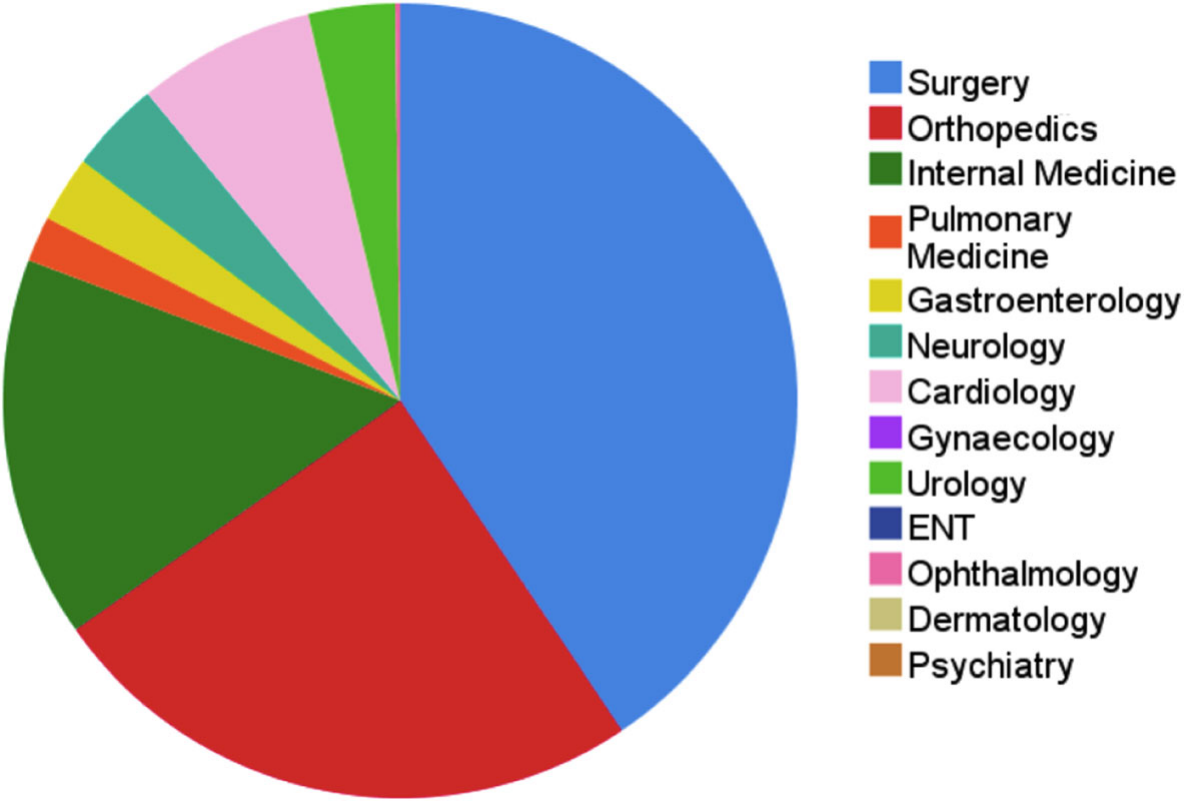
Pie chart of treating specialties

Of the 483 eligible patients who agreed to participate, 239 patients belonged to the control group and 244 patients to the intervention group (Fig. 1). The baseline characteristics are presented in Table 1. There were no differences noted in mean age, age category, gender, and complexity of the problem between the two groups. The only difference noted was one in education level. Figure 2 shows a pie chart of the treating specialties in both groups, in which surgery and orthopedics were the most common treating specialties.

**Table 1:** Baseline characteristics of the control and intervention group

	Control group	Intervention group	Overall	***p*** value
	*N* = 239	*N* = 244	*N* = 483	
	Mean (SD)	Mean (SD)	Mean (SD)	
	***N*** (%)	***N*** (%)	***N*** (%)	
**Age (years)**	52.41 (19.45)	49.95 (18.98)	51.17 (19.24)	0.160
Young adulthood (18–34)	49 (20.5)	64 (26.2)	113 (23.4)	0.275
Middle adulthood (35–64)	122 (51.0)	121 (49.6)	243 (50.3)	
Late adulthood (≥ 65)	68 (28.5)	59 (24.2)	127 (26.3)	
**Gender**				0.254
Male	112 (46.9)	127 (52.0)	239 (49.5)	
Female	127 (53.1)	117 (48.0)	244 (50.5)	
Education level*				0.047
Lower education	57 (23.8)	69 (29.0)	126 (26.4)	
Intermediate education	96 (40.2)	70 (29.4)	166 (34.8)	
Higher education	86 (36.0)	99 (41.6)	185 (38.8)	
Complexity of problem**				0.967
Low	112 (46.9)	112 (45.9)	224 (46.4)	
Medium	57 (23.8)	58 (23.8)	115 (23.8)	
High	70 (29.3)	74 (30.3)	144 (29.8)	
Peak time (yes)***	162 (67.8)	107 (43.9)	269 (55.7)	0.000

*6 missing education levels in the intervention group

**Low: fractures, contusions, wounds, hematomas; Medium: e.g., simple infections, kidney- or gallstones, stomach/rectal pain, bladder retention, hyperventilation; High: e.g., neurological (traumatic brain injury, headache), thromboembolic and cardiovascular diseases, multiple diagnoses

***2–7 PM, based on previous analysis of peak hours on our ED in the last 2 years

Of the 483 enrolled patients, 72 could not be reached by telephone at follow-up. The younger age category was slightly more represented in this group (Additional table 1).

Table 2 shows how often information was provided to the patient per domain. Though most domains were discussed in all conversations, only 63% of patients were informed about return precautions.

**Table 2:** Frequency of domains discussed during discharge conversation

Domains	Discussed during discharge conversation
	*N* (%)
1. Diagnosis	483 (100.0)
2. Treatment	482 (99.8)
3. Follow-up consultations	471 (97.5)
4. Return precautions	304 (62.9)
All domains discussed	295 (61.1)

Table 3 shows the baseline and follow-up scores of the control and intervention group in baseline and follow-up. Because not all domains were mentioned in all conversations, we calculated the difference in mean scores regardless of the number of domains discussed instead of a total score.

**Table 3:** Difference in mean score on the discussed domains between the control- and intervention group

	Control group	Intervention group	Mean difference	***p*** value	Effect size (Cohen’s d)
	Mean score (SD)	Mean score (SD)	(95% CI)		
**Baseline**	*N* = 239	*N* = 244			
Total baseline	3.72 (0.40)	3.95 (0.15)	0.23 (0.29, 0.18)	0.000	0.77
1. Diagnosis	3.78 (0.48)	3.97 (0.19)	0.19 (0.25, 0,12)	0.000	
2. Treatment	3.72 (0.57)	3.96 (0.20)	0.24 (0.32, 0.16)	0.000	
3. Follow-up consultations	3.83 (0.49)	3.96 (0.19)	0.13 (0.20, 0.07)	0.000	
4. Return precautions	3.40 (0.85)	3.90 (0.36)	0.50 (0.65, 0.36)	0.000	
**Follow-up**	*N* = 194	*N* = 217			
Total follow-up	3.68 (0.42)	3.85 (0.27)	0.17 (0.24, 0.10)	0.000	0.49
1. Diagnosis	3.76 (0.53)	3.90 (0.34)	0.14 (0.23, 0,05)	0.001	
2. Treatment	3.68 (0.61)	3.90 (0.33)	0.22 (0.32, 0.13)	0.000	
3. Follow-up consultations	3.85 (0.44)	3.92 (0.37)	0.07 (0.14, 0.02)	0.114	
4. Return precautions	3.13 (1.04)	3.60 (0.69)	0.47 (0.69, 0.26)	0.000	

The mean difference was 0.23 (95% CI 0.29, 0.18), corresponding with a 6.3% increase in score for the intervention group and an effect size of 0.77. The most prominent mean difference per domain was 0.50 for return precautions (95% CI 0.65, 0.36).

The table also shows the difference in scores at follow-up between both groups, where the mean difference is 0.17 (95% CI − 0.24, − 0.10) with a 4.5% increase in mean score in the intervention group and an effect size of 0.49. Once again, the domain of return precautions showed the most considerable difference. Though this domain also showed the most prominent decline in the longitudinal scores (− 0.30, 95% CI − 0.11, − 0.48, and − 0.31; 95% CI − 0.20, − 0.42; Additional table 2), still, more information was retained in the intervention group.

After adjusting for age, education level, complexity of the problem, presence of a partner, and peak time, an adjusted model shows that the mean scores were only significantly influenced by education level and the complexity of the problem (Additional table 3). A sensitivity analysis performed showed no effect of the number of discussed domains on the mean score (Additional table 4).

Table 4 shows the proportion of comprehension deficits across the four domains. In the control group, 49.0% of patients demonstrated a deficit in at least one domain compared to 11.9% in the intervention group. In the follow-up group this difference was still present but less prominent (53.1% vs 34.1%). In all groups, comprehension deficits were most frequent for return precautions.

**Table 4:** Proportion of patients with comprehension deficits

	Control group	Intervention group
	***N*** (%)	***N*** (%)
**Baseline**	(*N* = 239)	(*N* = 244)
Deficit in *at least* one domain	117 (49.0)	29 (11.9)
1. Diagnosis	45 (18.8)	6 (2.5)
2. Treatment	52 (21.8)	10 (4.1)
3. Follow-up consultations	31 (13.4)	9 (3.8)
4. Return precautions	59 (41.3)	13 (8.1)
**Follow-up**	(*N* = 194)	(*N* = 217)
Deficit in at least one domain	103 (53.1)	74 (34.1)
1. Diagnosis	37 (19.1)	18 (8.3)
2. Treatment	50 (25.8)	20 (9.2)
3. Follow-up consultations	23 (12.1)	13 (6.1)
4. Return precautions	56 (51.4)	43 (30.1)

Lastly, to assess feasibility, the duration of the teach-back conversation was measured compared to the discharge interview. The mean duration of a discharge interview was 3:11 min, whereas a teach-back conversation on average lasted an additional 1:39 min (Table 5).

**Table 5:** Duration of discharge and teach-back conversation

	Duration of discharge information (min:s)	Duration of teach-back conversation (min:s)
	*N* = 243*	*N* = 244
**Mean**	3:11	1:39
**Median**	2:46	1:25
**Minimum**	0:12	0:21
**Maximum**	14:51	8:51

*1 measurement failed

## Discussion

Our results show that the teach-back method can improve patient recall and comprehension of discharge information in all domains directly following ED discharge. After several days, an improvement can still be seen, but less than at discharge. In both the baseline and follow-up group, the greatest benefit of teach-back is seen with return precautions, in which deficits are most common. Nearly half of patients had incomplete comprehension after standard discharge, compared to one tenth of the patients after teach-back. We demonstrated that the teach-back conversation on average only adds less than 2 min to the complete discharge protocol of the ED patient. Therefore, it seems feasible to use in this method a busy ED setting. Lastly, we also saw that healthcare providers failed to provide relevant information, especially regarding return precautions, to more than one third of patients.

The positive effect of teach-back that we measured is largely similar as reported previously [15, 20]. Griffey et al. also showed an increased comprehension in post-ED care, including self-care and follow-up instructions. However, no significant difference was found on other domains [20]. Slater et al. showed the highest improvement of all on the diagnosis domain, but no significant increase in medication reconciliation [15]. Our study however shows an improvement in all four domains immediately after discharge. Several factors might explain this difference. First, only patients with a new medical problem were included in this study. When patients receive information about their problem for the first time, it might be more difficult for them to retain, and therefore more profit can be gained using the teach-back method. Second, the time of data collection differed; Griffey et al. collected data immediately after discharge and Slater et al. 6–30 h after discharge. Our study showed that after several days a reduction in knowledge is seen. Results may also be influenced by the complexity of the medical problem. As far as we know, ours is the first study that included this factor in recall scores and saw that patients with low complexity problems remembered their information better. Demographically, Slater et al*.* focused on all patients as we did but in an urban academic center, whereas Griffey et al. only focused on a population with low health literacy. Lastly, in our country—as in many others—it is not common practice to hand out written instructions or other adjuncts to patients discharged from the ED. Therefore, in our study, patients had to recall all information without using adjuncts during both the initial and delayed recall, whereas Slater et al. allowed patients to use additional written instructions [15].

The percentage of people leaving the ED with comprehension deficits in at least one domain in this study corresponds with the 41–78% found in previous studies [8, 9, 11–15]. Likewise, deficits most often concerned post ED care, including home care instructions and return precautions [11, 12, 15]. In general, patients are mostly only interested in the diagnosis and treatment, because these directly impact their health outcome. This might lead physicians to put more emphasis on those two domains and less on post ED care.

The findings regarding the percentage of insufficient information provided by physicians are similar to what was found in other studies (17–42%), including that return precautions are most often left out [8–10]. In our study, this outcome may to some extent be explained by the fact that part of our population had a fracture which required a cast. In these cases, the nurses, and not the physician, would explain the return precautions while applying the cast. Even when keeping this in mind, return precautions is still the domain that is left out most often. Time pressure and continuous interruptions in a busy ED are often named as factors that make effective communication in general more challenging. However, the fact that in case of incomplete information this is mostly due to return instructions may indicate that healthcare providers might be insufficiently aware of the importance of this domain. Patients on the other hand may not think of hypothetical situations in which they may need to return to the hospital and may not ask about it themselves.

The duration of the discharge conversation that we measured was similar to the findings of Marty et al. (2013), in which physicians spent an average of 4 min on the discharge conversation. Rhodes et al. (2004) however showed a duration of only 76 s [9, 24]. This difference could be explained by the fact that in some cases patients already received information during their stay in the ED that was not repeated at the discharge interview. The short duration of time needed to conduct a teach-interview might seem surprising at first. However, the discharge conversation itself on average takes 3–4 min. Taking into account that teach-back is merely a summary and repetition of this information given by the patient instead of the physician, it seems logical. Corrections that were often necessary were only part of that summary (e.g., taking medication three times instead of two times a day) and often did not take that much time either.

### Limitations

There were several limitations in this study. First of all, this was a single-center study that was neither randomized nor blinded. However, the demographic characteristics of the control and intervention group were still comparable. We tried to limit inter-observer variability by using standardized questions and clear guidelines on how to proceed in certain situations (e.g., when doctors did not mention certain domains during the discharge interview).

The Hawthorne effect may also have been a factor in this study, because both the patient and healthcare provider were aware of the observers during discharge. Therefore, both of them may have paid more attention to the content of the conversation, which could have led to a better-structured conversation and higher recall [25]. Though this might have led to overstated baseline scores, this should not have any influence on the difference in scores. The Hawthorne effect might also occur if a recording device would be used (as in the study of Griffey et al.). Extracting data from the medical record instead of observing the discharge conversation would eliminate this effect. However, this is a less reliable way to check the content of the discharge conversation, because in practice this often does not correlate well with the information verbalized. Often times, there is incomplete information written in the patient file and the ample use of medical jargon.

Second, as some eligible patients refused to participate, a non-responder bias may have influenced our results. Reasons to refuse participation could be long waiting times, feeling ill or tired, having the feeling of participating in a test, or the fear of making mistakes. This group probably possesses certain traits that could have led to lower mean scores. In addition, people with a language barrier were excluded in this study, leading to a selection bias, because these patients are at risk for not understanding discharge instructions [3].

Our study supports previous findings and shows the effectiveness of teach-back on for patients’ short-term recall and comprehension. Future research should include testing the teach-back method’s long term impact, including complications, return ED visits, and re-admissions. Further research is also required to assess the acceptability of teach-back among healthcare providers. One other subject that remains to be explored is if teach-back is also an effective approach for patients with language barriers, as these patients are more at risk of comprehension deficits.

## Conclusion

Information provided to patients discharged from the emergency department is often incomplete or insufficient, especially concerning return precautions. The information that is given is often not comprehended completely or recalled correctly. In our study, we found that the teach-back method significantly increases recall and comprehension of discharge information in adult ED patients with a new diagnosis. With the important finding that a teach-back conversation on average takes less than 2 min, this method could potentially be easily incorporated into any general ED practice guideline.

## Supplementary information

**Additional file 1: Table S1.** Demographic characteristics of the completed and loss to follow-up group.

**Additional file 2: Table S2.** Mean difference between patients’ immediate recall and recall at follow-up.

**Additional file 3: Table S3.** Linear regression analysis to correct for possible confounding in baseline and follow-up group.

**Additional file 4: Table S4.** Sensitivity analysis comparing mean score of patients who received information on all four domains to patients who received incomplete information.

## Data Availability

The datasets used and/or analyzed during the current study are available from the corresponding author on reasonable request.
