# Supplementary material for: The impact of teach-back on patient recall and understanding of discharge information in the emergency department: the Emergency Teach-Back (EM-TeBa) study
Source: Int J Emerg Med. 2020 Sep 24;13:49. doi: 10.1186/s12245-020-00306-9 (PMC7513274; doi:10.1186/s12245-020-00306-9)
Supplement: Supplementary file 1 — Additional file 1: Table S1. Demographic characteristics of the completed and loss to follow-up group. [file 12245_2020_306_MOESM1_ESM.docx]

**Additional table 1**

**Demographic characteristics of the completed and loss to follow-up group**

|  | | **Completed follow-up group** | **Loss to follow-up group** | **Overall** | **p-value** |
| --- | --- | --- | --- | --- | --- |
|  | | N = 411 | N = 72 | N = 483 |  |
|  | **Mean (SD)** | **Mean (SD)** | **Mean (SD)** |  |  |
| **Age (Yrs)** | | 51.74 (18.80) | 47.88 (21.42) | 51.17 (19.24) | 0.116 |
|  | | **N (%)** | **N (%)** | **N (%)** |  |
| - Young adulthood (18-34)  - Middle adulthood (35-64)  - Late adulthood (≥65) | | 87 (21.2)  215 (52.3)  109 (26.5) | 26 (36.1) 28 (38.9)  18 (25.0) | 113 (23.4)  243 (50.3)  127 (26.3) | 0.017 |
| **Gender (%)**  - Male  - Female | | 202 (49.1%)  209 (50.9%) | 37 (51.4%)  35 (48.6%) | 239 (49.5)  244 (50.5) | 0.726 |
| **Education level (%)***  - Lower education  - Intermediate education  - Higher education | | 105 (25.7)  145 (35.5)  158 (38.7) | 21 (30.4)  21 (30.4)  27 (39.1) | 126 (26.4)  166 (34.8)  185 (38.8) | 0.626 |
| **Complexity of problem**  - Low  - Medium  - High | | 194 (47.2)  94 (22.9)  123 (29.9) | 30 (41.7)  21 (29.2)  21 (29.2) | 224 (46.4)  115 (23.8)  144 (29.8) | 0.488 |
| **Peak time (Yes)**** | | 226 (55.0) | 43 (59.7) | 269 (55.7) | 0.456 |

* 3 missing educational levels in both groups

** 2-7PM
