# Supplementary material for: The impact of teach-back on patient recall and understanding of discharge information in the emergency department: the Emergency Teach-Back (EM-TeBa) study
Source: Int J Emerg Med. 2020 Sep 24;13:49. doi: 10.1186/s12245-020-00306-9 (PMC7513274; doi:10.1186/s12245-020-00306-9)
Supplement: Supplementary file 2 — Additional file 2: Table S2. Mean difference between patients’ immediate recall and recall at follow-up. [file 12245_2020_306_MOESM2_ESM.docx]

**Additional table 2 Mean difference between patients’ immediate recall and recall at follow-up**

|  | Immediate recall | Recall after 2-4 days | Mean difference (95% CI) | p-value |
| --- | --- | --- | --- | --- |
| Control group  N = 194 | Mean (SD) | Mean (SD) |  |  |
| All discussed domains  1. Diagnosis  2. Treatment  3. Follow-up consultations  4. Return precautions | 3.75 (0.38)  3.80 (0.47)  3.74 (0.54)  3.85 (0.42)  3.43 (0.89) | 3.68 (0.42)  3.76 (0.53)  3.68 (0.61)  3.85 (0.44)  3.13 (1.05) | 0.07 (0.01, 0.12)  0.04 (-0.03, 0.10)  0.06 (-0.02, 0,15)  0.00 (-0.07, 0.06)  0.30 (0.11, 0.48) | 0.015  0.275  0.107  0.879  0.002 |
| Intervention group  N= 217 |  |  |  |  |
| All discussed domains  1. Diagnosis  2. Treatment  3. Follow-up consultations  4. Return precautions | 3.95 (0.15)  3.97 (0.19)  3.95 (0.21)  3.96 (0.20) 3.91 (0.33) | 3.85 (0.27)  3.90 (0.34)  3.90 (0.33)  3.92 (0.37)  3.60 (0.69) | 0.10 (0.07, 0.13)  0.07 (0.02, 0.12)  0.05 (0.01, 0.10) 0.04 (-0.00, 0.09)  0.31 (0.20, 0.42) | 0.000  0.007  0.018  0.060  0.000 |
