# Supplementary material for: The impact of teach-back on patient recall and understanding of discharge information in the emergency department: the Emergency Teach-Back (EM-TeBa) study
Source: Int J Emerg Med. 2020 Sep 24;13:49. doi: 10.1186/s12245-020-00306-9 (PMC7513274; doi:10.1186/s12245-020-00306-9)
Supplement: Supplementary file 3 — Additional file 3: Table S3. Linear regression analysis to correct for possible confounding in baseline and follow-up group. [file 12245_2020_306_MOESM3_ESM.docx]

**Additional table 3**

**Linear regression analysis to correct for possible confounding in baseline and follow-up group**

| **Baseline** | | **Unstandardized Coefficients** | **Standardized Coefficients** | |  | **p-value** |
| --- | --- | --- | --- | --- | --- | --- |
|  |  | **B** |  | **Beta** |  |  |
|  | (Constant) | 3.88 |  |  |  | 0.000 |
|  | Study group | 0.23 |  | 0.36 |  | 0.000 |
|  | Patient age | -0.00 |  | -0.05 |  | 0.282 |
|  | Education level | 0.04 |  | 0.11 |  | 0.021 |
|  | Complexity of problem | -0.05 |  | -0.14 |  | 0.001 |
|  | Peak time | -0.02 |  | -0.03 |  | 0.456 |
|  | Presence of partner | 0.00 |  | 0.00 |  | 0.950 |
| **Follow-up** | |  |  |  |  |  |
|  | (Constant) | 3.62 |  |  |  | 0.000 |
|  | Study group | 0.17 |  | 0.24 |  | 0.000 |
|  | Patient age | -0.00 |  | -0.11 |  | 0.026 |
|  | Education level | 0.04 |  | 0.09 |  | 0.077 |
|  | Complexity of problem | -0.09 |  | -0.22 |  | 0.000 |
|  | Presence of partner | 0.02 |  | 0.03 |  | 0.550 |

* Dependent variable: mean score
