# Supplementary material for: The impact of teach-back on patient recall and understanding of discharge information in the emergency department: the Emergency Teach-Back (EM-TeBa) study
Source: Int J Emerg Med. 2020 Sep 24;13:49. doi: 10.1186/s12245-020-00306-9 (PMC7513274; doi:10.1186/s12245-020-00306-9)
Supplement: Supplementary file 4 — Additional file 4: Table S4. Sensitivity analysis comparing mean score of patients who received information on all four domains to patients who received incomplete information. [file 12245_2020_306_MOESM4_ESM.docx]

**Additional table 4**

**Sensitivity analysis comparing mean score of patients who received information on all four domains to patients who received incomplete information**

|  | **Complete information provided** | **Incomplete information provided** | | **p-value** |
| --- | --- | --- | --- | --- |
|  | **Mean score (SD)** | **Mean score (SD)** |  | |
| **Control group** | 3.68 (0.40) | 3.76 (0.40) | 0.136 | |
| **Intervention group** | 3.95 (0.16) | 3.96 (0.12) | 0.745 | |
| **Overall** | 3.83 (0.33) | 3.85 (0.32) | 0.413 | |
